# Advancing cell therapies for intervertebral disc regeneration from the lab to the clinic: Recommendations of the ORS spine section

**DOI:** 10.1002/jsp2.1036

**Published:** 2018-10-08

**Authors:** Lachlan J. Smith, Lara Silverman, Daisuke Sakai, Christine L. Le Maitre, Robert L. Mauck, Neil R. Malhotra, Jeffrey C. Lotz, Conor T. Buckley

**Affiliations:** ^1^ Department of Neurosurgery University of Pennsylvania Philadelphia Pennsylvania; ^2^ Department of Orthopaedic Surgery University of Pennsylvania Philadelphia Pennsylvania; ^3^ Translational Musculoskeletal Research Center Corporal Michael J. Crescenz VA Medical Center Philadelphia Pennsylvania; ^4^ DiscGenics Inc. Salt Lake City Utah; ^5^ Department of Neurosurgery University of Tennessee Health Science Center Memphis Tennessee; ^6^ Department of Orthopaedic Surgery, Surgical Science Tokai University School of Medicine Isehara Japan; ^7^ Biomolecular Sciences Research Centre Sheffield Hallam University Sheffield UK; ^8^ Department of Bioengineering University of Pennsylvania Philadelphia Pennsylvania; ^9^ Department of Orthopaedic Surgery University of California San Francisco California; ^10^ Trinity Centre for Bioengineering Trinity Biomedical Sciences Institute, Trinity College Dublin, The University of Dublin Dublin Ireland; ^11^ School of Engineering Trinity College Dublin, The University of Dublin Dublin Ireland; ^12^ Advanced Materials and Bioengineering Research (AMBER) Centre Royal College of Surgeons in Ireland & Trinity College Dublin, The University of Dublin Dublin Ireland

**Keywords:** biological therapies, biomaterials, preclinical models, stem cells

## Abstract

Intervertebral disc degeneration is strongly associated with chronic low back pain, a leading cause of disability worldwide. Current back pain treatment approaches (both surgical and conservative) are limited to addressing symptoms, not necessarily the root cause. Not surprisingly therefore, long‐term efficacy of most approaches is poor. Cell‐based disc regeneration strategies have shown promise in preclinical studies, and represent a relatively low‐risk, low‐cost, and durable therapeutic approach suitable for a potentially large patient population, thus making them attractive from both clinical and commercial standpoints. Despite such promise, no such therapies have been broadly adopted clinically. In this perspective we highlight primary obstacles and provide recommendations to help accelerate successful clinical translation of cell‐based disc regeneration therapies. The key areas addressed include: (a) Optimizing cell sources and delivery techniques; (b) Minimizing potential risks to patients; (c) Selecting physiologically and clinically relevant efficacy metrics; (d) Maximizing commercial potential; and (e) Recognizing the importance of multidisciplinary collaborations and engaging with clinicians from inception through to clinical trials.

## 1 INTRODUCTION

Lower back pain is a leading cause of disability worldwide and the third most expensive health condition in the United States, with estimated health care spending in excess of $80 billion annually.^1^, ^2^ It is a significant epidemiological and socioeconomic problem affecting quality of life, and is the most common, non‐cancer reason for opioid prescription in the United States.^3^ Intervertebral disc degeneration is a progressive, cell‐mediated cascade of molecular, structural and biomechanical changes, which is strongly implicated as a cause of “discogenic” back pain.^4^ The incidence of disc degeneration is linked to aging, trauma, genetics, lifestyle and the presence of co‐morbidities.^5^, ^6^, ^7^, ^8^, ^9^ The intervertebral discs are the pliant, fibrocartilaginous joints required for transferring compressive loads and supporting complex mobility of the spine.^10^ Each disc is comprised of a central, proteoglycan‐rich, gelatinous nucleus pulposus (NP), a peripheral, fibrocartilaginous annulus fibrosus (AF), and superiorly and inferior, hyaline cartilage end plates that interface with the vertebral bodies.^10^ The earliest degenerative changes typically manifest in the NP, where reductions in proteoglycan content and hydration compromise biomechanical function, leading to progressive degeneration of the entire intervertebral joint.^11^, ^12^ Self‐repair and regeneration of the NP is limited by a low cell density and a limited nutrient supply.^13^, ^14^ Conventional treatments for disc degeneration are focused solely on alleviation of symptoms and often have limited long‐term efficacy.^15^, ^16^, ^17^ This is exacerbated by the lack of an accepted clinical standard for discogenic pain, where clinicians are often unable to identify a specific nociceptive cause.^18^, ^19^ Because of this, the rationale for choosing surgery for these patients is controversial. Spinal fusion does not restore natural biomechanical function and has been shown to induce adjacent segment pathology (ASP) caused by increased mechanical stress in adjacent segments.^20^ More specifically, in a study comparing 725 lumbar fusion cases to 725 randomly selected controls with chronic low back pain diagnoses, focusing on the outcome return to work (RTW) revealed only 26% of patients had RTW 2 years after fusion surgery, while 67% of nonsurgical controls had RTW within 2 years from the date of injury.^21^ In addition, of the patients receiving spinal fusion, 36% had complications and the reoperation rate was 27%, with 66% of the reoperations occurring within 2 years of the index surgery. Of the 36% of complications, failed and/or implant malposition represented 4.7% of cases, while late spinal complications such as disc space infection, pseudarthrosis, postlaminectomy syndrome, adjacent disc degeneration, stenosis, spondylolisthesis, and adjacent vertebral fracture represented 25.2% of cases, with the balance relating to early major systemic, neurologic and wound complications.

There is therefore a tremendous unmet need for new treatment options for patients with disc degeneration and associated lower back pain. To address this need, there has been significant recent interest in developing injectable cell‐based therapies for the treatment of disc degeneration with the specific aim to stimulate tissue repair.^22^ Such therapies represent a potentially low risk and low cost long term solution for a very large patient population, making them attractive from both clinical and commercial standpoints.

The pathway to bringing new cell therapies to patients involves extensive in vitro testing, pre‐clinical validation in small and large animal models, and human clinical trials to demonstrate safety, efficacy, and commercial viability. Successful translation involves multidisciplinary engagement and interactions between clinicians, scientists, engineers and industry. Clinicians are particularly important as they can provide access to primary human cells, advise on patient selection criteria and outcome metrics, assist with the design and conduct of clinical trials, support risk/value discussions with regulatory and reimbursement agencies, and advocate the treatment to patients and health systems.

Even after demonstrating success in animal studies, cell‐based therapies are at risk of stalling in the “valley of death”, which comprises multiple significant barriers that include scale‐up, manufacturing, regulatory, business and market obstacles. To effectively move cell therapies out of the lab and into the clinic, the following must be considered and addressed: (a) Optimizing cell sources and delivery techniques; (b) Minimizing potential risks to patients; (c) Selecting physiologically and clinically relevant efficacy metrics; (d) Working with industry and maximizing commercial potential; and (e) Recognizing the importance of forming multidisciplinary collaborations and engaging with clinicians from inception through to clinical trials. The focus of this article is to highlight and comprehensively review each of these critical areas, and in doing so provide a set of recommendations for successfully advancing cell‐based disc therapies from the lab to the clinic for first‐in‐human studies.

## 2 CURRENT COMMERCIAL APPROACHES FOR CELL‐BASED DISC REPAIR AND REGENERATION

A small number of companies have successfully entered the clinic to evaluate the efficacy of cell‐based disc regeneration treatments in humans. These treatments have employed a variety of cell types including those obtained from both homologous (from the disc) and non‐homologous (eg, from knee cartilage, adipose tissue) sources, and have utilized both autologous and allogeneic approaches. Autologous Disc Cell Transplantation (ADCT) by Co.Don AG (Berlin, Germany) has been in clinical use for the treatment of degenerate discs for a number of years,^23^ with recent investigations demonstrating that re‐implantation of culture‐expanded NP cells can retard degenerative changes in patients with herniated discs^24^; however, there are limitations associated with ADCT delivery and efficacy, including cell leakage following injection into the disc,^25^ diminished tissue forming capacity of culture expanded NP cells derived from degenerated tissue^26^ and the paucity of NP cells that can be isolated. The challenge of cell leakage is not unique to ADCT per se, and is a function of the delivery and surgical techniques employed. Any cell therapy relying on intradiscal needle delivery in a saline solution will likely suffer similar failings unless it is combined with some form of biomaterial (eg, hydrogel, microcarriers, microcapsules), which can enhance cellular retention in the disc space.

Two companies have disclosed results from initial clinical trials and have subsequently progressed to performing additional clinical trials. ISTO Biologics (St. Louis, Missouri) reported promising results from a Phase I study evaluating allogeneic juvenile chondrocytes mixed with a fibrin carrier,^27^ known as NuQu; however the Phase II study was recently terminated for undisclosed reasons (clinicaltrials.gov study identifier NCT01771471). Mesoblast Ltd. (Melbourne, Australia) also showed promising results using immunoselected adipose‐derived mesenchymal stem cells (MSCs) injected into the discs of patients, including finding that the treatment resulted in pain relief and improved water content in treated discs compared to controls.^28^, ^29^ When injected into degenerate ovine discs, these cells were shown to elicit regenerative effects.^30^ A larger, multi‐national Phase III study is ongoing to better assess the potential efficacy and safety of this treatment (clinicaltrials.gov study identifier NCT02412735). Biorestorative (Melville, New York) has evaluated BRTX‐100, an autologous stem cell therapy, in a preliminary human clinical study and has investigational new drug (IND) approval to start a trial in the United States (no clinicaltrials.gov information available).

A number of other companies are currently executing first‐in‐human clinical trials. Utilizing cells modified from adult disc tissue, DiscGenics Inc. (Salt Lake City, Utah) is conducting a clinical trial to evaluate safety and efficacy of the product candidate IDCT (injectable discogenic cell therapy) compared to controls over 2 years (clinicaltrials.gov study identifier NCT03347708). IDCT contains a mixture of specialized therapeutic progenitor cells engineered from donated adult disc tissue, or “discogenic” cells and a viscous carrier material. Promising animal data suggests a potential therapeutic benefit of IDCT, including disc height improvement and normalization of disc architecture, and a safe profile.^31^ Aesculap Biologics (Breinigsville, Pennsylvania) is currently testing NOVOCART DISC in a Phase II trial in the European Union. This treatment is a combination of autologous disc chondrocytes and a self‐polymerizing hydrogel (clinicaltrials.gov study identifier NCT01640457) that is already being used clinically for articular cartilage repair in the knee. Preliminary results from the Phase I trial of this therapy indicate the risks of adverse events occurring are comparable to elective disc surgery.^32^

In summary, commercial efforts to date have applied an extensive variety of cell‐based approaches for treating disc degeneration. While some of these have shown encouraging preclinical and clinical findings with respect to both safety and efficacy, none have achieved widespread clinical adoption.

## 3 PATIENT SELECTION: EVEN THE BEST THERAPY WON'T SUCCEED IF APPLIED TO THE WRONG DISC

For effective clinical translation it will be important to stratify patient cohorts, for example using sophisticated magnetic resonance imaging techniques^33^, ^34^, ^35^, ^36^, ^37^ and quantification of circulating biomarkers^38^ to select suitable candidates for treatment. The opportunity for cell‐based therapy may be most appropriate at the early stage of the degenerative cascade, prior to the onset of advanced disc degeneration coupled with endplate calcification and extensive annular degeneration.^39^ As a first step, patient selection for cell‐based therapies should therefore be limited to single‐level, moderate severity disc degeneration, which is reflected by Pfirrmann Grade III or IV on MRI. Patients presenting with a Pfirrmann grade of II or V may represent a patient population either too mild or too advanced in the disease process to show measurable improvement.^40^

Currently, patient selection for intradiscal therapies includes chronic back pain (>4 VAS) and disability (>40 ODI) that is refractory to conservative care (longer than 6 months). Potential patients are excluded if there is evidence of clinically‐relevant vertebral/spinal abnormalities that include spondylolisthesis, spondylolysis, scoliosis, fracture, severe kyphosis, or disc herniation. Herniation patients may be suitable candidates for treatment if intradiscal therapies are applied in combination with AF repair. Within appropriate individuals, the offending level(s) is typically identified by imaging (MRI) plus other clinical criteria that may include invasive techniques such as provocative discography. While current imaging techniques can provide exquisite anatomic detail, standard MRI sequences do poorly at identifying specific pain generators.^41^ Recently, a group of international experts concluded that there is no accepted clinical standard for discogenic pain.^18^, ^19^, ^42^, ^43^ Equally important, treatment outcomes are currently judged by subjective and qualitative patient reports, which can be influenced by significant placebo effects.^44^ Therefore, to support clinical application and evaluation of intradiscal cellular therapies, there is a critical need for new techniques that localize pain generators and quantify therapeutic effects on treated levels. New, advanced imaging tools for this purpose are currently in development and have been summarized in several recent reviews.^45^, ^46^ Examples include MRI techniques such as T1ρ and T2 relaxation times, and chemical exchange saturation transfer (CEST), which, unlike more widely used subjective grading disc grading schemes, are quantitative surrogates for disc composition.

## 4 OPTIMIZING CELL SOURCES AND DELIVERY TECHNIQUES

### 4.1 The challenges of the degenerate disc microenvironment

The rationale for and benefits of delivery of cells to the NP are 2‐fold: firstly, transplanted cells may stimulate endogenous NP cells to produce neo‐matrix through paracrine effects; and secondly transplanted cells may adopt an NP cell‐like phenotype and directly reconstitute native tissue.^47^, ^48^ A central challenge, however, that limits the potential of cell‐based regeneration is the harsh local cellular microenvironment within the degenerate disc, which is characterized by low oxygen and nutrient supply, increased acidity, altered osmolarity, as well as elevated levels of pro‐inflammatory cytokines.^49^, ^50^, ^51^, ^52^, ^53^, ^54^ Therefore, there is a clear need to identify robust cell populations to enhance the likelihood of survival post‐injection, and characterize how such cells will function in the typical degenerate microenvironment to determine if they can contribute effectively to functional disc repair. The precise cell number for intradiscal delivery needs to be carefully considered, and to‐date the exact number of cells required for functional regeneration is unclear. This will most likely depend on the cell‐type being employed as well as the metabolic activity and percentage survival post intradiscal delivery. A recent review of clinical trials by Sakai and Schol reported cell numbers ranging from 1 to 40 million cells being used in investigations.^55^ It is postulated that the delivery of large numbers of cells may exacerbate the degenerative microenvironment due to competing nutrient demands, which may in turn result in ineffective clinical outcomes.^56^ Given the varying stages of degeneration and microenvironments that exist in vivo, it will be important to tailor or design treatments for individuals. One way to achieve this could be to condition stem cells prior to implantation by acclimatizing them to patient specific in vivo microenvironmental milieu, which may include inflammatory cytokines, acidity, oxygen or nutrient deprivation.^57^, ^58^, ^59^ These treatments must be assessed using appropriate in vitro and ex vivo culture conditions which mimic those of the degenerate niche to assess the likely behavior and survival of transplanted cells prior to progressing to in vivo testing.^49^

### 4.2 Primary cells, stem cells or gene therapy?

From a surgical and practical perspective, given the unique structure and location of the disc, the difficulty in obtaining autologous primary NP tissue and cells for therapeutic use from either herniated discs or adjacent intact levels is clear. Delivery of primary NP cells to the disc appears safe and has shown some potential for efficacy in early clinical trials.^60^ Additional limitations of primary cells include the diminished tissue forming capacity of culture expanded NP cells derived from degenerated tissue^26^ and the altered catabolic phenotype of such cells, together with the paucity of NP cells that can be isolated. Furthermore, the isolation of tissue from adjacent healthy disc levels may increase the risk of initiating degeneration at the harvest site.^61^ This has motivated many researchers into identifying and characterizing alternative cell sources for disc regeneration, which is a key step towards translating therapies into clinical practice.^62^ Primary, differentiated cells from other skeletal sites with reduced risk for donor site morbidity, such as articular and nasal cartilage, have been investigated in vitro and in animal models for NP regeneration.^63^, ^64^, ^65^ While these cell types do exhibit some phenotypic differences to native NP cells, their relative ease of isolation, differentiated state and higher propensity to deposit extracellular matrix may make them attractive alternatives in the future and warrant further consideration and exploration. The likelihood that these alternative cell types can adopt a true discogenic phenotype is remote, and whether the extracellular matrix constituents they deposit are suitable to provide biomechanical functionality and longevity to restore disc function remains to be fully established.

Stem cell therapies have received considerable attention due to their versatility, translatability and potential for long term native tissue regeneration. Some successful patient outcomes have been demonstrated in clinical studies using this approach.^28^, ^29^, ^55^, ^66^, ^67^, ^68^ The bone marrow has been the prime site of MSC isolation for disc regeneration applications, inclusive of in vitro, preclinical and clinical studies.^61^, ^69^ Progenitor cells derived from other tissue sources such as adipose tissue have also been shown to possess significant potential for differentiation and tissue forming capabilities.^70^, ^71^ Adipose derived stem cells (ADSCs) may provide a better alternative and candidate for cell therapy and disc regeneration, due to their abundance and ease of isolation, and may also have a lower inherent capacity than bone marrow derived MSCs to undergo endochondral ossification.^72^ Harvesting of autologous ADSCs can be performed in outpatient clinics with typical yields of up to 25 000 adherent ADSCs per gram of tissue.^73^ One of the potential challenges in utilizing stem cells is that they are undifferentiated. The precise mechanism of action for regeneration is unknown; whether injected stem cells have the capacity to differentiate towards a discogenic phenotype or stimulate resident native cells has not been clearly demonstrated in preclinical studies. Priming stem cells using growth factors or other environmental cues such as altered oxygen or glucose availability to direct or maintain a specific phenotype may enhance their regenerative potential,^58^, ^59^, ^74^, ^75^, ^76^ but may also add complexity to the regulatory approval process.

Notochordal cell‐based approaches for disc regeneration have received considerable interest in the past several years. Notochordal cells are the progenitors of adult NP cells, and their loss in humans during postnatal growth is thought to contribute to the onset of degeneration later in life.^10^ As notochordal cells themselves cannot be readily obtained for direct clinical application, most studies have focused on leveraging the therapeutic potential of notochordal cell‐secreted factors.^77^

Gene therapy approaches may hold significant promise for disc regeneration, for example through silencing of catabolic or activating anabolic pathways. Gene therapy has advantages over direct delivery of proteins or small molecules, among them the possibility of sustained efficacy through long‐term, regulated, endogenous synthesis of growth factors or anti‐inflammatory factors.^78^ However, the need to establish a safety profile and possible off target effects will most likely lead to a longer and more complex regulatory process before realizing a commercially available product and clinical translation.

### 4.3 Effective cell delivery and retention

With respect to delivery of cell‐based therapies, the primary mode of choice has been injection through the AF. A key translational advantage of this approach is that, with image‐guided assistance, it can be performed percutaneously. However, there are associated challenges that have the potential to compromise both safety and efficacy, including the potential for needles to induce permanent AF damage,^79^ cell leakage from the delivery site and diminished cell viability due to shear forces as cells are forced through small diameter needles.^80^, ^81^, ^82^ For example, injecting a cell suspension into the lumbar discs of rabbits resulted in a 90% loss of the injected cells within the first 30 minutes.^83^ Biomaterials as delivery vehicles, and in particular hydrogels, have been utilized to overcome retention issues and provide additional support for cell survival and phenotype retention.^84^, ^85^, ^86^, ^87^, ^88^, ^89^ Injectable biomaterial systems utilizing alginates, collagens, hyaluronic acid, fibrin and a variety of other substances have all shown promise for improving cell delivery for disc repair^90^, ^91^, ^92^; however, for clinical translation, the biomaterial itself will require regulatory approval. Using biomaterials already approved and in clinical use for other indications may accelerate the approval process. That being said, because the disc is avascular, carrier degradation products may accumulate to levels beyond those found in other target tissues, justifying disc‐specific toxicity testing. Selection of needle size for trans‐annular delivery should be a careful trade‐off between the minimum size necessary to prevent shear‐induced cell death and allow injection of viscous hydrogels, and the maximum size necessary to prevent permanent AF damage and/or cell leakage. In a recent study using a preclinical large frame goat model, a 22G needle was found not to induce discernable degenerative changes on MRI or histology after 12 weeks.^93^ In addition, the volume to be delivered needs to be optimized, as excessive volumes may result in over pressurization, increasing the likelihood of cell leakage upon needle withdrawal. Further, the spatial distribution of delivered cell populations may also impact cell differentiation or activity and subsequent regeneration outcomes, as injected materials will tend to preferentially concentrate within pre‐existing fissures that are common in degenerated discs. For hydrogel‐delivered cell populations, migration of cells through the NP and integration of the injected material with native tissue should be considered. An alternative delivery route that has been investigated, and which does not cause disruption to the AF, is through the pedicles/endplates (transpedicular approach);^94^, ^95^ however, whether such an approach will have detrimental effects to the integrity of the endplate in the long term is yet to be established.

## 5 MINIMIZING POTENTIAL RISKS TO PATIENTS

Demonstrating safety is critical for disc therapies, as the consequences of an adverse event are potentially severe. The disc is one of the most highly loaded tissues in the body, which creates the significant potential that injected biomaterials will migrate in response to long term, cyclic loading. Material that is expelled from the disc space may impinge on nerves and result in neurological complications or even paralysis. Therefore, a fundamental safety requirement of a disc therapy, particularly one that involves a structural component such as a hydrogel or scaffold, is that it structurally integrates, and remains completely and permanently contained within the disc space upon delivery. Retention should be verified in the presence of physiological loading; ex vivo, this can be accomplished through extensive cyclic and multiaxial loading. Long term in vivo retention can be assessed via incorporation of radiopaque contrast agents or nanoparticles at the time of delivery, facilitating non‐invasive and three dimensional longitudinal assessments.^85^

Ensuring that a therapy does not impart local or systemic toxicity is also a critical safety consideration, and in vivo biocompatibility studies are generally a requirement of the FDA (food and drug administration) and other regulatory bodies for new biological therapies. Toxicity could potentially result directly from the implanted material, or over time from its degradation by‐products. For example, biomaterials that break down into acidic by‐products in the confined space of the disc could contribute to eliciting a catabolic, local, cellular response.^96^ Toxicity and biocompatibility can be assessed in vitro through cellular co‐culture studies and tissue or organ culture studies, and in vivo via implantation subcutaneously as well as in the disc space itself. Outcome measures from in vivo studies may include assessing the viability of endogenous cells and recruitment of neutrophils, macrophages, mast cells and fibroblasts, and the extent of fibrous encapsulation at the site of implantation, which can provide indications of an inflammatory response. Monitoring for signs of systemic toxicity (acute and chronic) is also essential, through assessment of changes in behavior, weight and appetite, reflexes and sensitization, in addition to blood chemistry, hematology and anatomic pathology.

Prior to clinical testing, stem cell therapies must be carefully vetted in vitro and through preclinical studies. Genetic instability of undifferentiated, multi‐ or pluripotent cells poses a safety risk as it increases the chances of tumorigenesis and cancer.^97^ Pluripotent stem cells such as embryonic and induced pluripotent stem cells exhibit intrinsic genetic instability that can result in teratoma formation.^97^ Emerging non‐integrating techniques for cellular reprogramming, for example using synthetic mRNA or chemical methods, may mitigate these risks without compromising induction efficiency.^98^, ^99^ Adult, multipotent stem cells, while not exhibiting the same tumorigenic risk as pluripotent stem cells, may also exhibit genetic instability through chromosomal aberrations that manifest progressively with increasing numbers of expansion passages.^97^ Minimizing the length of culture and degree of cellular manipulation prior to implantation minimizes the propensity for these in vitro genetic alterations to occur. Assessing the heterogeneity of stem cell populations in culture is also important to reduce tumorigenic and immunogenic risk.

Gene therapy, which employs cellular reprogramming, poses unique safety challenges that may limit or complicate translatability.^100^ These risks, which include the potential for insertional mutagenesis and cancer, and antigenicity with associated adverse immunological responses, result in additional regulatory hurdles, which may be harder to overcome for therapies targeting non‐life‐threatening conditions such as disc degeneration. Risks associated with gene therapy may be mitigated through careful selection of gene delivery systems (eg, viral vs non‐viral), which minimize risk without comprising efficacy.^101^

The method and frequency of administration will also impact a therapy's safety profile. Therapies that can be delivered using minimally invasive procedures, such as image‐guided percutaneous injection, and do not require general anesthesia, will pose less risk to patients than therapies that require an open surgical approach. Examples of such therapies might include injection of stem cells, hydrogels or drugs directed at the NP. Other strategies, such as implantation of cell‐seeded engineered constructs to repair or replace degenerate disc tissue, may necessitate an open surgical approach, however the required surgical procedures should carry no greater risk than those in current clinical use. Similarly, therapies that require a single administration to achieve long term efficacy will have a more attractive safety profile than those that require multiple administrations.

## 6 SELECTING PHYSIOLOGICALLY AND CLINICALLY RELEVANT EFFICACY METRICS

### 6.1 Defining and understanding the clinical problem

Optimization of disease‐modifying activity should be informed by knowledge of the patient, their clinical needs, and underlying pain and disability mechanisms. The most important objective of a treatment for painful disc degeneration is long term (>6 months) alleviation of symptoms. Current treatment strategies, whether they be conservative or surgical, may be effective in providing short term relief from pain, but their long‐term efficacy is more problematic, in part because they do not seek to restore disc structure or mechanical function. Given the nature of disc degeneration and its cascading effect on adjacent tissues and overall spine mechanical properties, it is often difficult to specify the fundamental treatment target. Disruption of normal disc biomechanical properties is associated with accelerated degeneration of other structures such as facet joints, and osteophyte formation due to adaptive bony changes or calcification of ligaments.^102^, ^103^ Back pain patients commonly have associated leg pain and symptoms which are frequently secondary to nerve compression, due to stenosis caused by loss of disc height and/or bulging or herniation. Consequently, stabilization (if not restoration) of disc structure is a key goal.

Back pain can also arise from irritation of peripheral disc tissues. For example, pro‐inflammatory crosstalk between the disc and vertebra is thought to underlie bone marrow edema evidenced as Modic changes (MC) on clinical MRI.^104^ Cellular expression of cytokines (eg, IL‐1, −6, and −10, and RANKL)^104^, ^105^ with MC is linked to endplate erosions which strongly associate with back pain.^106^, ^107^ These observations indicate that inflammation suppression is another important therapeutic goal.

For cell therapies to be clinically adopted, they need to demonstrate benefits beyond current treatment options. The rationale for choosing between surgical and nonsurgical care for chronic low back pain patients is not well‐defined. While the benefit of surgery for “mechanical” back pain (such as instability and sciatica) is supported by significant outcome literature, the appropriate intervention for disc‐related pain is less clear. Fusion surgery may alleviate pain by restoring intervertebral height and decompressing nerves, but immobilizes the intervertebral joint.^15^, ^17^ Metal/polymer‐based total disc arthroplasties preserve a limited degree of joint mobility, but are subject to wear and potential failure requiring revision surgery, and have not seen widespread adoption.^108^ Reported success of surgical care for back pain ranges from 41% to 57%,^109^ and with 5% to 16% early complication and reoperation rates.^110^ Discectomy may alleviate symptoms by removing herniated or bulging disc material and decompressing nerves, but the damaged disc is not repaired and may continue to degenerate, and many patients continue to experience pain and require ongoing treatment.^111^ Non‐surgical approaches such as steroid injections and physical therapy are temporary treatments that seek to mask symptoms that might otherwise resolve over time. Recurrent treatment for unresolving symptoms is more problematic as permanent nerve damage can occur. Non‐surgical, like surgical, treatment does not alter the long term progression of degeneration. Restoration of disc biomechanical function is therefore key to effective long‐term alleviation of painful symptoms.

The primary attraction of biological, cell‐based disc regeneration therapies is that they have the potential to alleviate symptoms *and* stabilize disc structure as well as biomechanical function by reconstituting native tissue structures. When delivered in combination with a structural biomaterial such as a hydrogel or composite scaffold, cell‐based therapies can provide immediate normalization of disc structure and mechanical function, while bioactive elements such as cells or growth factors work in vivo to suppress inflammation and progressively replace the biomaterial with native tissue. The three most important objectives to demonstrate efficacy of biological disc regeneration therapies are therefore alleviation of pain, stabilization or restoration of structure, and normalization of biomechanical function. Outcome measures for in vitro, in vivo preclinical, and clinical evaluation of such therapies should be selected with these goals in mind.

### 6.2 Appropriate selection and implementation of model systems to demonstrate efficacy

**Figure 1:**
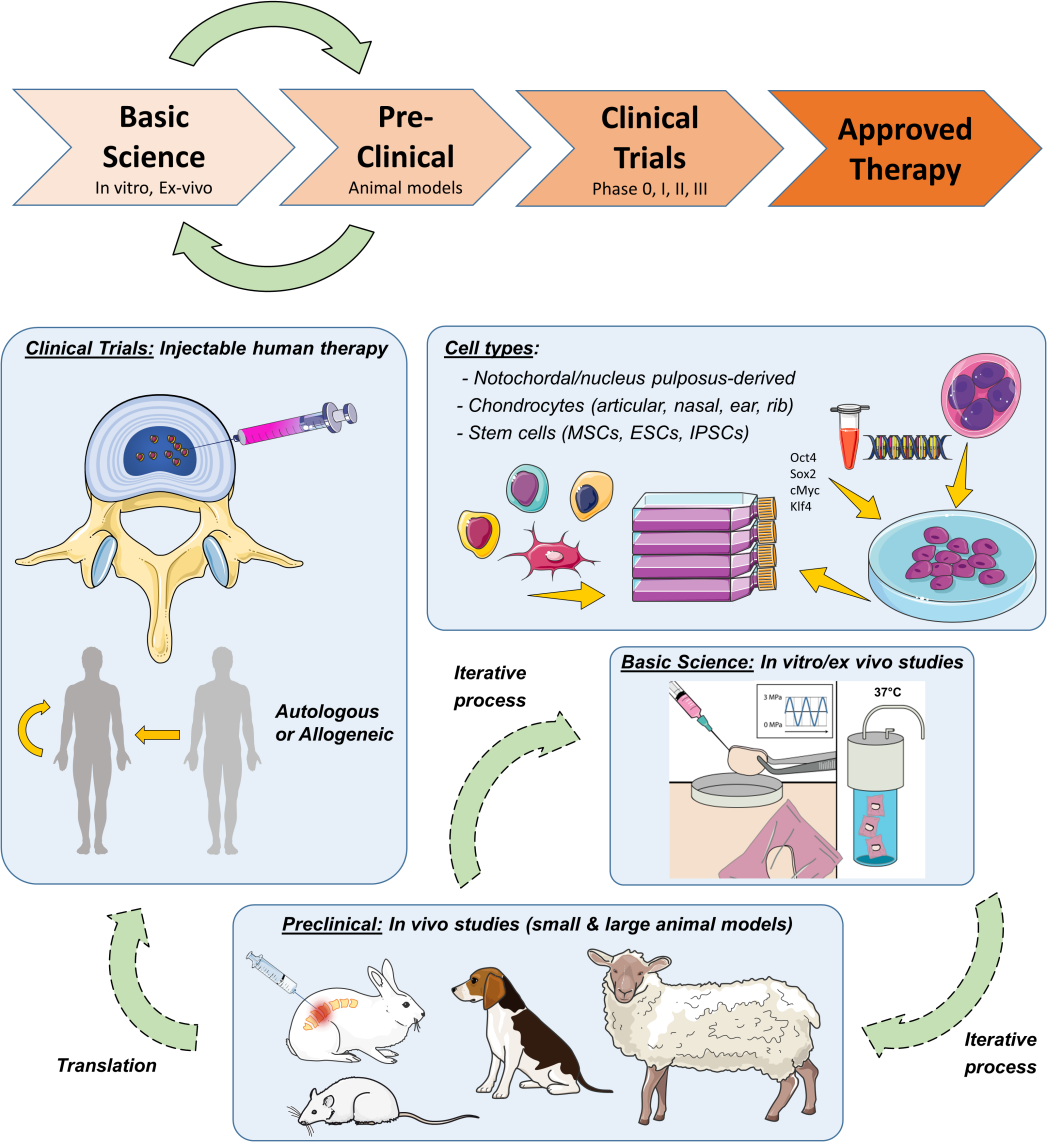
Requisite steps for demonstrating the efficacy of cell‐based disc regeneration therapies. Model systems should be applied iteratively, balancing experimental control, cost and throughput in the early stages with biological complexity and clinical relevance at more advanced stages, in order to maximize the chances of success in the clinic

Pre‐clinical demonstration of efficacy and safety using in vitro and in vivo model systems is an iterative process (Figure 1), and typically begins with simple two‐dimensional (2D) or three‐dimensional (3D) cell culture models that have limited biological complexity but are cost effective and high throughput. Experiments may then progress to more complex 3D culture models or organ culture systems that incorporate more elements of the in vivo cellular environment. Smaller animal models such as mice, rats or rabbits may then be used to establish preliminary in vivo efficacy, and immunocompromised small animal models enable preliminary in vivo evaluation of human cells. Preclinical evaluations may then be conducted using larger animals such as pigs, sheep, goats or dogs. The reason for such an iterative approach to demonstrating therapeutic efficacy reflects the need to balance experimental control, cost and throughput on the one hand during proof‐of‐concept, with the need to incorporate biological complexity and demonstrate long term efficacy on the other, to progress towards clinical translation. At a minimum, a broad understanding of the biological mechanisms underlying cell‐based regeneration strategies is required to achieve efficacy, both for hypothesis generation at the study outset and to direct troubleshooting and optimization.

In order to maximize the physiological relevance of experimental data, it is important that model systems effectively recapitulate the in vivo cellular microenvironment of the degenerate human disc. If therapeutic efficacy is evaluated only under idealized experimental conditions, the chances of failure upon preclinical or clinical translation are high. While this is true to an extent for therapies targeting any number of conditions (ie, not just disc degeneration), the disc microenvironment poses unique challenges—biochemical, biophysical and biomechanical—to therapeutic cell survival and function. With respect to the biochemical environment, as the disc has little or no direct blood supply, the cells must survive and function with access to very limited oxygen and nutrition. As discs degenerate, even in the early stages, this biochemical environment is further characterized by increasing local catabolic cytokine expression^112^ and acidity.^113^ Therapeutic cell types such as MSCs are more sensitive to microenvironmental stressors such as oxygen, nutrient deprivation and inflammatory stimuli compared to endogenous cells.^114^, ^115^, ^116^

For in vitro cell culture models, mimicking this in vivo disc microenvironment can be accomplished by culturing cells in low oxygen, and reduced glucose and serum, to simulate nutrient deprivation.^115^, ^116^, ^117^ The degenerate environment can be further simulated by including catabolic mediators such as inflammatory cytokines and by increasing acidity.^118^, ^119^, ^120^ The physical environment can be mimicked through culturing in soft 3D scaffolds such as hydrogels. Mechanical stress can be simulated using bioreactors that apply dynamic compressive loads and/or hydrostatic pressure.^121^, ^122^, ^123^, ^124^ Organ culture systems that employ living, degenerate discs from human donors may be the ultimate platform to effectively mimic the biological complexity of the in vivo cellular microenvironment, particularly when combined with a bioreactor that applies physiological loading.^125^

Ensuring accurate recapitulation of the human disc cellular microenvironment is equally important when moving to in vivo systems. One of the greatest challenges facing in vivo models of disc therapeutics is the need to replicate the size, and in particular the height of the human disc, necessary to accurately recapitulate physiological nutritional demands. Large animals obviously more closely approximate the nutritional environment compared to rodents, however, even in livestock, lumbar disc size is still significantly smaller than in humans (eg, lumbar disc height in sheep is ~4 mm compared to ~11 mm in humans).^126^

Therapeutic cell types such as MSCs may exhibit different phenotypic characteristics as a function of donor, age, species and anatomical site, and these characteristics may be closely linked to regenerative potential.^127^, ^128^, ^129^, ^130^ While the development phase may necessitate the use of non‐human cells that are accessible and compatible with in vitro and in vivo models, the results obtained with these cells may not accurately reflect the performance of human cells that will be used clinically. It is therefore important to validate findings using cells from human donors, wherever possible controlling for variables such as age, sex, co‐morbidities and anatomical site. Human cells may be obtained both from commercial sources, as well as from clinical collaborators undertaking spinal procedures. The latter of these sources is particularly attractive where it permits recruitment of donors who would also be prospective recipients of disc therapeutics. For example, a surgeon performing a procedure such as a spinal fusion may be able to provide not only surgical waste tissue as a source of endogenous disc cells, but also bone marrow from the adjacent vertebrae without subjecting the patient to any additional surgical procedures or risks. As already discussed, organ culture using degenerate human discs obtained from cadavers represents an avenue for evaluating therapeutic efficacy not only using human cells, but also in a physiologically accurate and degenerative microenvironment. The importance of cell source also extends to the preclinical development phase. For example, a therapy that demonstrates efficacy in vitro using cells of a particular age and species, may not demonstrate similar efficacy in a large animal model of a different age and species. In vitro studies should therefore seek to validate a therapeutic approach using cells from the full range of sources that will be required at each phase of development.

### 6.3 Pain and disability outcome measures

As alleviation of pain is the primary goal of disc therapies, including pain as an outcome measure in laboratory experiments is desirable. Selection of physiologically‐relevant pain outcome measures in the preclinical setting is complicated, as in the clinical setting pain is subjective and its direct source may be ill‐defined.^131^, ^132^ Pain outcome measures can be classified in different ways. They may include surrogates such as nerve ingrowth and regression,^133^, ^134^ local and systemic levels of secreted neurogenic and inflammatory factors,^135^, ^136^ as well as imaging to demonstrate nerve compression.^45^ Direct outcome measures of pain may include assessments of overall behavior and mobility, and pain sensitivity assays.^137^ Small animal models are particularly attractive with respect to assessing pain, as they are compatible with most if not all of these outcome measure types. For example, if a therapy is being evaluated in a mouse or rat, it is possible and practical to assess mobility, pain sensitivity, and serum and radiological biomarkers in vivo, and tissue level expression of neurogenic markers post mortem. Using in vitro models, pain outcomes are limited to surrogates such as expression of neurogenic factors and nerve ingrowth. Large animal models are in theory compatible with many of the same outcome measures as small animal models, but practical considerations make implementation of functional and pain sensitivity assays more challenging.

### 6.4 Structural and biomechanical outcome measures

Including appropriate outcome measures to assess restoration of disc structure and mechanical function is equally important, as these are the key indicators of the likelihood for a therapy to provide long term alleviation of symptoms. Like pain, the ability to assess these parameters directly or indirectly is dependent on the type of model system. With respect to structure, the most clinically relevant outcome measure is stabilization or restoration of disc height as demonstrated through imaging (MRI, plain radiographs or computed tomography), and is possible for in vivo models as well as in vitro whole‐disc organ culture models.^93^, ^138^, ^139^ Importantly, these non‐invasive imaging metrics can be applied longitudinally and thus used to confirm both acute regenerative effects and sustained, long term efficacy. With respect to biomechanical properties, in vitro model systems that incorporate three‐dimensional constructs or whole‐disc organ culture facilitate direct assessment of mechanical function.^89^, ^140^ For in vivo models, mechanical properties can be assessed ex vivo on intact spinal motion segments or isolated tissue samples.^85^ In the case of motion segments, multi‐axis loads can be applied to similar physiological deformations, including those likely to lead to failure.^141^ Direct, in vivo evaluation of disc mechanical properties is possible using emerging technologies such as magnetic resonance elastography.^142^, ^143^ Where direct evaluation is impossible, assessments of extracellular matrix composition and organization through biochemical and histological assays, or imaging (eg, MRI or contrast‐enhanced CT), represent important surrogates for mechanical function.^33^, ^34^, ^35^, ^36^, ^37^

## 7 MAXIMIZING COMMERCIAL POTENTIAL

Given the vast socio‐economic costs associated with low back pain and the high costs of current treatment approaches, it would appear, superficially, to be straight forward to establish the commercial case for a new disc therapy, particularly one for which clinical safety and efficacy can be established. However, bringing a biological disc therapy to market is a long, complex and expensive process, and one that to date has been met with little success. Basic scientists, including those working in the translational space, are often focused foremost on probing basic mechanistic questions and rapidly disseminating findings. They may be less concerned about addressing questions of commercial viability, despite this being an essential criterion for widespread clinical adoption. A new biological therapy may “check” key boxes with respect to safety and efficacy in vitro, in animal models, and even in clinical trials, but fail at the final hurdle if it is not perceived as commercially viable. Some of the factors impacting potential commercial viability can be addressed early on, including clearly identifying the prospective target patient population size, protecting intellectual property, and assessing the competitive landscape. Other factors should be considered and continuously reviewed as the concept evolves, including complexity, cost of components and preparation time, in addition to the ease and method of administration. Early and frequent discussions with regulators (eg, FDA) are key to defining the studies required to translate such products into the clinic, and to ensure that manufacturing approaches will meet the standards required for human testing.

One predictor of the potential commercial viability of a new therapy is the size of the prospective target patient population. Further, the overall manufacturing costs associated with producing the product, in contrast to the reimbursement/pricing potential of the treatment, must be favorable. In considering the clinical motivation for developing a specific biological disc therapy, the overall socioeconomic burden is often cited, as is the current lack of effective treatment options. In establishing and justifying the clinical need for a specific, new therapeutic approach, it is necessary to move beyond general patient populations (ie, “degenerative disc disease”) and stratify subgroups of patients that will be suitable candidates for that therapeutic approach based on the specific disease features experienced. For example, injectable disc therapies may be tailored to target a patient subgroup that exhibits a specific set of symptoms or a narrow window of degeneration severity that includes an intact annulus and relatively healthy endplates. The need for a therapy targeting symptomatic low back pain in patients with mild to moderate severity disc degeneration (eg, minimally invasive cell therapy) could be justified by citing the number of patients in this subgroup that are not candidates for surgery and are unresponsive to conservative treatment approaches. Alternatively, the need for a new therapy targeting patients with severe disc degeneration (eg, tissue engineered total disc replacements) could be justified by citing the number of patients that exhibit poor outcomes from current surgical approaches such as fusion or total disc arthroplasty.

The second important consideration to ensure future commercial viability is early protection of relevant intellectual property. At most academic institutions, protection of intellectual property begins by submitting an invention disclosure to the relevant university office, which will then assess the concept or technology for commercialization potential. Such disclosures are a requirement for any potentially patentable invention, and institutions may stipulate that this should be done in advance of any public disclosure (eg, publication of manuscripts or conference presentations). Without such protection before public disclosure, the ownership is lost, and no patent may be filed, significantly decreasing the chances of the product ever being translated to the clinic. Following disclosure, where an invention is identified as having commercialization potential, the institution should be equipped to guide the investigator through the intellectual property protection process, including filing of patents. Trade secrets represent an alternative approach to patenting an idea, though not one necessarily supported by academic institutions. When a patent is published and becomes public, smart competitors have the opportunity to circumvent it by implementing minor alterations or improvements to the overall concept. In the case of trade secrets, key properties of the product that are critical to its function never enter the public domain.

The relative cost and complexity of a therapeutic strategy, with respect to production, preparation and administration will also impact commercial viability. A complex and costly in vitro approach may be necessary during early stages of development; however, investigators should consider how this can be reduced as the development process advances towards preclinical and clinical translation. This process of refinement might include minimizing (or eliminating) the number of growth factors, cell types or biomaterial components, substituting growth factors with cheaper compounds, and reducing the time needed for in vitro manipulation prior to administration, which may include cell expansion or conditioning.

For first or second generation therapeutic products, investigators should also be cognizant and take steps at an early stage to avoid developing “complex” approaches, which will most likely face significant regulatory hurdles and barriers. For example, this might include considering using autologous vs allogeneic cells, or using appropriately conditioned and sourced adult stem cells vs pluripotent cells that require viral reprogramming. Therapies that require *in vitro* manipulation using animal serum face tough regulatory hurdles and alternative culture conditions should be considered. If the approach includes gene therapy, then gene delivery systems with the best safety profile should be selected. The number of regulatory hurdles could be further reduced by incorporating or substituting compounds that have an established safety record and are already FDA approved for other indications.

The final important consideration with respect to ensuring commercial viability is to undertake a comprehensive survey of the current competitive landscape. This requires identifying competing therapeutics, including those in clinical use and those undergoing clinical or preclinical trials, that target a similar cohort of patients, and reviewing where those competing products are with respect to the development pipeline. If a competing product has not already come to market, it should be estimated when that is likely to happen. Even if the number of competing products is relatively small, factors such as relative risk, cost and potential efficacy compared to the therapy being proposed should be assessed.

## 8 THE IMPORTANCE OF MULTIDISCIPLINARY COLLABORATION AND ENGAGING WITH CLINICIANS

Closely linked to commercial viability and prospective market uptake is the willingness of clinicians to embrace the newly developed therapeutic approach. There may be resistance within a particular market sector to switch away from traditional treatment approaches, for example, for philosophical, financial or logistical reasons. To ensure a new disc therapy will successfully and effectively translate from the lab to the clinic, basic scientists must therefore work to ensure that the new product will ultimately be enthusiastically embraced by those clinicians required to administer it. To achieve this, it is essential to engage clinicians early and continuously from inception through to translation, and these clinical collaborators may well already be passionate about identifying novel treatment strategies for their low back pain patients. Not only will the participation of clinicians (as the end users) aid market acceptance, it will also add significant value to the overall scientific endeavor. In the early stages, clinicians will be able to draw on their extensive, firsthand experience to help identify prospective target patient populations, and advise on existing treatment modalities and their limitations. As development progresses through *in vitro* evaluations, clinical collaborators can provide access to primary human cells, for example from surgical waste tissue, that can be used to maximize the physiological relevance of results. During preclinical evaluation in animal models, clinical collaborators can work to optimize application of the treatment, surgically or otherwise, advise on selection of clinically relevant outcome measures and evaluate success benchmarks. As a therapy approaches clinical translation, clinical collaborators can disseminate and promote preclinical findings to colleagues at clinical scientific meetings, assist with the design and conduct of clinical trials, including recruitment of trial participants, navigate the regulatory landscape, and advocate the treatment to patients and health systems. It is thus crucial that clinical collaborators fully understand the character of the product and underlying science.

Engagement with clinicians can also be enhanced through dissemination of research findings not only at major basic science‐intensive meetings such as the Orthopedic Research Society (ORS), but also at meetings that have stronger clinical participation, such as those of the International Society for the Study of the Lumbar Spine (ISSLS), the Cervical Spine Research Society (CSRS), the North American Spine Society (NASS), the American Academies of Orthopedic (AAOS) and Neurological (AANS) surgeons. Clinicians are frequent and active participants in these meetings, and constitute leadership positions, and are thus the ideal advocacy vehicles in these fora. Equally, it is pivotal to promote and encourage multidisciplinary interactions at both scientific and clinically focused meetings to accelerate the field towards clinical translation and ensure we maximize our efforts in treating degenerative disc disease.

## 9 SUMMARY

Despite an overwhelming clinical need, cell‐based therapies for disc degeneration and low back pain have to date failed to achieve widespread clinical adoption. Over the last decade, tremendous advances have been made towards understanding the underlying pathophysiology, defining and optimizing therapeutic cell types, and refining and applying physiologically relevant model systems for their evaluation. The goal now must be to more effectively translate laboratory findings into the clinic to achieve improved patient outcomes. To this end, the primary intention of this perspective from members of the Orthopedic Research Society Spine Section is to provide a consensus with respect to the central challenges that have limited effective clinical translation of cell‐based therapies. Motivating this perspective was a uniform and sincere belief among Spine Section members that cell‐based therapies, if effectively designed and implemented in conjunction with appropriate diagnostic tools, patient selection criteria, and with the support of industry partners have the potential for substantial clinical impact. Recommendations for researchers to effectively address these challenges are provided, falling broadly under the key themes of “Safety, Efficacy, Commercial Viability and Engaging Clinicians” (summarized in Table 1). The authors urge researchers to consider and leverage these recommendations to enhance the translational relevance and clinical potential of their research endeavors and activities.

**Table 1:** Key recommendations for successful clinical translation of injectable cell‐based disc therapies

Safety	Efficacy	Commercial viability	Engaging clinicians
Demonstrate long term retention in the disc space under physiological loading	Select experimental outcomes relevant to the overall goal of long term alleviation of symptoms through stabilization/ restoration of disc structure and biomechanical function	Identify the size of the prospective target patient population	Engage clinicians early and continuously during development
Evaluate acute and chronic, local and systemic toxicity in vitro and in vivo, and include effects of degradation products	Apply model systems iteratively, balancing experimental control, cost and throughput in the early stages with biological complexity and clinical relevance at more advanced stages	Protect intellectual property early by submitting an invention disclosure to the relevant university office	Anticipate resistance to switching away from current therapeutic approaches
Minimally invasive delivery carries less risk than an open surgical approach	Design model systems to appropriately mimic aspects of the in vivo chemical, physical and mechanical microenvironments of the disc	Minimize cost and complexity with respect to production, preparation and administration	Clinicians can provide primary human sourced material for in vitro testing, and develop and refine techniques for in vivo modeling and therapeutic administration
Aim for a single administration vs multiple administrations	Consider the confounding effects of species, age and sex on efficacy in vitro and in animal models	Minimize the number of potential regulatory hurdles	Clinicians can advise and lead design and implementation of clinical trials, including patient recruitment
Maximize genetic stability of stem cell therapies by minimizing in vitro manipulation	Incorporate human‐sourced cells and tissues into experiments	Undertake a comprehensive survey of the competitive landscape	Clinicians can advocate new therapeutic approaches to patients and health systems, accelerating their early and widespread adoption
Autologous cell therapies pose less of a risk than allogeneic	Where outcomes such as pain or mechanical properties cannot be measured directly, carefully justified, clinically relevant surrogates should be used		Clinicians can facilitate dissemination and promotion of new therapeutic approaches to colleagues at clinical scientific meetings
	Clearly define success benchmarks for all experimental outcomes		

## CONFLICT OF INTEREST

Drs Smith, Buckley, Sakai, Mauck and Malhotra have no conflicts of interest to declare. Dr Silverman is an employee of DiscGenics, Inc. Dr Le Maitre is a named inventor on a patent for an injectable hydrogel system (GB2493933). Dr Lotz is a founder of Relivient Medsystems and Nocimed.

### Author contributions

All authors contributed to the writing of the manuscript. Drs Smith and Buckley designed the outline and revised the manuscript, and all authors read and approved the final version prior to submission.
